# Technologies for Proteome-Wide Discovery of Extracellular Host-Pathogen Interactions

**DOI:** 10.1155/2017/2197615

**Published:** 2017-02-22

**Authors:** Nadia Martinez-Martin

**Affiliations:** Department of Microchemistry, Proteomics and Lipidomics, Genentech, South San Francisco, CA, USA

## Abstract

Pathogens have evolved unique mechanisms to breach the cell surface barrier and manipulate the host immune response to establish a productive infection. Proteins exposed to the extracellular environment, both cell surface-expressed receptors and secreted proteins, are essential targets for initial invasion and play key roles in pathogen recognition and subsequent immunoregulatory processes. The identification of the host and pathogen extracellular molecules and their interaction networks is fundamental to understanding tissue tropism and pathogenesis and to inform the development of therapeutic strategies. Nevertheless, the characterization of the proteins that function in the host-pathogen interface has been challenging, largely due to the technical challenges associated with detection of extracellular protein interactions. This review discusses available technologies for the high throughput study of extracellular protein interactions between pathogens and their hosts, with a focus on mammalian viruses and bacteria. Emerging work illustrates a rich landscape for extracellular host-pathogen interaction and points towards the evolution of multifunctional pathogen-encoded proteins. Further development and application of technologies for genome-wide identification of extracellular protein interactions will be important in deciphering functional host-pathogen interaction networks, laying the foundation for development of novel therapeutics.

## 1. Introduction

The plasma membrane constitutes a critical biological interface between the cytosol and the extracellular environment of the cell, and consequently membrane-tethered proteins and secreted molecules (collectively termed extracellular proteins) are essential regulators of cellular communication. From high affinity cytokine-receptor interactions to low affinity cell-cell adhesion contacts, extracellular protein-protein interactions (ePPIs) are key for information processing and coordination of virtually all processes in a living organism. Furthermore, given their fundamental functions and their accessibility to systemically delivered drugs, extracellular proteins are particularly suitable targets for therapeutic intervention. In fact, despite these proteins being encoded by approximately one-fourth of the human genes, at least two-thirds of the existing drugs target either secreted or membrane-bound proteins [1]. Thus, the elucidation of the ePPI networks on a global scale has become crucial for the biomedical research. However, in spite of their relevance and abundance, ePPIs are remarkably underrepresented in available large-scale datasets. This discrepancy is due to the low sensitivity and limited compatibility of current high throughput technologies to detect extracellular interactions because of the unusual biochemical nature of the membrane proteins and the intractability of their binding partners [2–4]. In particular, transmembrane domain-containing proteins are amphipathic, making it difficult to solubilize them in their native conformation, and often contain posttranslational modifications such as glycans and disulfide bonds, which are not properly added in common heterologous expression systems [5]. In addition, interactions between cell surface proteins are often characterized by fast dissociation rates and therefore weak binding affinities, and in consequence well-established PPI methods such as yeast-two-hybrid or affinity purification-mass spectrometry (AP/MS) largely fail to detect these interactions. Over the last decade, several innovative technologies have been developed to overcome the aforementioned technical challenges and allow for sensitive detection of ePPIs [2, 6–10]. Nevertheless, the mapping of ePPIs remains a major challenge in biology.

Infectious diseases result in millions of deaths each year and therefore identifying new candidate targets for improved therapeutic development remains a pressing health concern. Pathogens have evolved a myriad of elegant and often complex strategies to invade the host and commandeer host immune responses to allow pathogen replication, spread, and persistence in the infected organism. Many cell surface molecules serve as entry receptors for initial host cell invasion, and concerted responses to the pathogenic challenge critically rely on cell functions mediated by receptors and secreted proteins. To allow host colonization, pathogens encode highly optimized protein modulators, in the form of secreted molecules or receptors expressed on the plasma membrane of the infected cells or the surface of the pathogen [11, 12]. Interactions between these proteins and extracellular host molecules form the foundation of communication between a host and a pathogen and play a vital role in the initiation and outcome of the infection [13, 14]. Characterizing host-pathogen ePPI networks is therefore of utmost importance to gain a better understanding of the infection process and to inform the development of novel or improved therapeutic strategies. Excellent studies on mapping host-pathogen interactions, particularly MS-based analysis of viral infection, have provided a wealth of insight into infectious diseases [15–19]. Nevertheless, similarly to host ePPIs, a significant hurdle to the elucidation of host-pathogen biology has been the shortage of datasets of extracellular interactions between host and pathogen proteins, partly due to the technical challenges that these proteins present. Moreover, an additional consideration when studying pathogen-encoded molecules is that these proteins often lack any recognizable homology with any host molecules, thus precluding prediction of their functions [11, 20]. Robust methodologies that permit unbiased characterization of ePPI in the absence of preexisting hypotheses are thus needed to elucidate the binding partners and molecular functions of most pathogen-encoded molecules.

Excellent reviews have recently revisited the currently available technologies for proteome-wide ePPI discovery [4, 8, 53–55]. Here we discuss the application of some of these technologies to the study of host-pathogen interaction and describe some of the major findings that have recently impacted research in the field of extracellular host-pathogen recognition. Protein microarrays and functional genomics approaches are highlighted here as emerging techniques with unique potential for the elucidation of host-pathogen ePPI networks at a genome-wide scale.

## 2. Biochemical and Biophysical Approaches

Classical biochemical and biophysical approaches are particularly suitable for detection of high affinity host-pathogen interactions, such as those mediated by a viral capsid protein and a host cell surface receptor, or a pathogen-encoded glycoprotein and a secreted host factor. Typically, these approaches have relied on the utilization of recombinant pathogen proteins as baits to probe for host binding partners, followed by immunoprecipitation and MS, or biophysical techniques for analysis of PPI such as surface plasmon resonance (SPR). SPR requires prior knowledge of the possible interacting partners and is therefore unsuitable for unbiased PPI discovery, whereas immunoprecipitation and MS approaches usually fail to detect weak interactions, which often characterize ePPIs, particularly those that take place on the cell surface. Notwithstanding, the identification of the receptors for some of the most prominent pathogens, such as the severe acute respiratory syndrome coronavirus (SARS-CoV) or the New World Arenaviruses, was made utilizing standard immunoprecipitation techniques [56, 57]. Despite their inherent limitations, biochemical approaches continue to provide relevant insights into host-pathogen interactions, such as the discovery of the dipeptidyl peptidase 4 (DPP4) as the receptor for the MERS-CoV just within a few months after the emergence of this virus [58]. In addition, more recently Kabanova and colleagues identified the cell surface receptor for the trimeric entry complex gHgLgO encoded by human cytomegalovirus (hCMV) [21]. Several studies have shown that the gHgLgO trimer is involved in the infection of fibroblasts, whereas the gHgLUL128L pentameric complex is required for entry into endothelial, epithelial, and myeloid cells [59–61]. In this work, both the trimeric and the pentameric CMV protein complexes were generated as recombinant products and used as baits to perform binding experiments on biotinylated cell surfaces, followed by immunoprecipitation and MS identification of bait-cell receptor complexes. Using this approach, the authors identified the cell surface protein PDGFR*α* as a high affinity receptor for the gHgLgO trimer and demonstrated that this interaction was required for infection of fibroblasts. Interestingly, in the case of the pentameric CMV complex, multiple bands were detected upon protein immunoprecipitation from epithelial cells, suggesting the existence of multiple receptors on these cells, which so far remain unknown [21] (Table 1).

Different biophysical techniques for detection of PPI, in particular SPR, have also proven valuable in the field of host-pathogen interactions. SPR offers the advantage of label-free, sensitive detection of interactions between a diversity of ligands in real time, thus allowing calculation of kinetic parameters. SPR has been widely utilized to monitor antibody binding to a variety of pathogen antigens, information that has informed vaccine development [62–64]. The SPR technology has also been exploited for discovery of ePPIs. For example, Viejo-Borbolla and coworkers utilized SPR to screen several secreted and membrane-expressed glycoproteins encoded by herpes simplex viruses (HSVs) for binding to chemoattractant cytokines (the chemokine family) and were able to identify a subset of human chemokines that bound to HSV glycoprotein G with high affinity [22]. Recently, Day and colleagues utilized a combination of glycan arrays and SPR and identified over 60 host-bacterial glycan pairs characterized by a wide range of binding affinities, some of which participated in bacterial adherence to host cells in vitro, leading to the hypothesis that bacteria-host surface glycan interactions may mediate initial attachment to the target cell during infection [65]. Despite SPR and related methods offering higher sensitivity for detection of transient PPIs than most biochemical approaches, these biophysical techniques have not been exploited for large-scale ePPI discovery, possibly due to the low throughput of the available instrumentation and the overall difficulties for generation of the relevant protein libraries.

These studies, among many others, have demonstrated the power of the classical biochemical and biophysical techniques for detection of host-pathogen interactions. Nevertheless, these approaches require previous knowledge of the pathogen-encoded proteins responsible for binding and the ability to produce such proteins as recombinant reagents, which may be challenging, as exemplified by the production of hCMV entry complexes [21, 66]. Alternative methods have been utilized in those cases where there is no previous knowledge of the pathogen proteins required for interaction with the host cells. In this regard, the screening of large collections of monoclonal antibodies raised against membrane proteins has proven particularly useful to identify receptors that mediate viral entry. Back in the early 80s, the discovery of CD4 as an entry receptor for the human immunodeficiency virus type 1 (HIV) significantly impacted our understanding of viral pathogenesis and subsequent development of therapeutics [23, 24]. In this case, the well-defined tropism of the virus determined the choice of over 100 antibodies directed against human leukocyte differentiation antigens, of which only antibodies that recognized the surface receptor CD4 blocked viral infection [23]. It is worth noting that similar monoclonal antibody screens have also been utilized for unbiased characterization of viral blockers. For example, Colonno and colleagues performed a screen of more than 2,000 hybridomas from mice immunized with preparations of plasma membranes from human cells and were able to find one antibody that blocked rhinovirus binding to its cell surface receptor [25], identified as the intercellular cell adhesion molecule 1 (ICAM-1) in subsequent studies [26].

Despite the undoubted importance of the biochemical and biophysical approaches to the study of host-pathogen interactions, the aforementioned limitations have motivated the development of alternative technologies for large-scale analysis of ePPIs.

## 3. Protein Microarrays

From the initial utilization of microarrays for detection of PPI over a decade ago, human proteome chips containing thousands of recombinant proteins have been generated, some of which are now commercially available. Protein microarrays offer the unique advantage of requiring minimal consumption of protein reagents, fast readouts, and relatively more affordable instrumentation. Typically, a fluorescently labeled or tagged protein of interest (the bait) is generated as a recombinant product and screened against all proteins in the array [10, 53]. Despite the increased availability of high-coverage protein arrays, very few are focused on extracellular proteins and therefore are not suitable for study of host-pathogen ePPIs. Most existing microarray-based methodologies rely on multimerization of the bait protein for increased avidity and detection of weak ePPIs, mimicking the way these interactions occur in vivo, where proteins are arrayed in the crowded molecular environment of apposing plasma membranes. Different multimerization strategies and protein microarray libraries have been developed and utilized for host-pathogen interaction discovery, some of which are described in more detail below.

### 3.1. Avidity-Based Extracellular Interaction Screen (AVEXIS)

The Wright lab developed a novel method for detection of low affinity ePPIs, termed avidity-based extracellular interaction screen (AVEXIS) [2]. In brief, AVEXIS consists of the expression of the extracellular domain (ECD) of the bait of interest as a recombinant protein, which retains its binding properties while removing the insoluble transmembrane region of the protein. These ECDs are tagged with a coiled-coil sequence from the rat cartilage oligomeric matrix protein to allow for pentamerization of the bait and therefore increased binding avidity, alongside a *β*-lactamase tag for detection of bait-prey interactions upon incubation with the colorimetric substrate nitrocefin. This multivalent strategy has been used for medium-scale screens, allowing detection of weak interactions between human receptors with low false-positive rates [2]. Notably, Crosnier and colleagues applied AVEXIS to search for the plasma membrane receptor responsible for* Plasmodium falciparum* infection of erythrocytes [27]. The authors compiled a library consisting of most secreted or cell surface-expressed proteins in erythrocytes and systematically assayed more than 40 red blood cell proteins for binding to* P. falciparum* protein PfRh5, a parasite protein essential for blood stage growth, expressed as an AVEXIS pentameric bait. Notably, the Ok blood group antigen BASIGIN was identified as a unique receptor for PfRh5, and inhibition of this interaction was shown to be sufficient to block parasite invasion of the erythrocyte, findings that may importantly inform antimalarial therapies [27]. In later studies, AVEXIS was miniaturized making this approach compatible with the protein microarray format, thus permitting more comprehensive and lower resource-intensive screenings [67]. Although this technique should allow for high throughout and sensitive determination of ePPIs, this approach has not yet been applied to elucidation of pathogen-host interactions.

### 3.2. Extracellular Protein Microarray Platforms

Over a decade ago, fueled by the recent completion of the human genome, Genentech pioneered a significant effort to identify novel secreted or transmembrane domain-containing proteins, upon careful bioinformatics assessment and high throughput protein purification [68]. These efforts resulted in the generation of a comprehensive human protein library, which was subsequently utilized to develop an extracellular protein microarray platform, consisting of over 1,500 secreted or single-transmembrane domain containing proteins [10]. For the generation of this human protein library, secreted proteins or the ECD of single-transmembrane receptors were fused to different affinity tags and subsequently purified from cell culture supernatants by size-exclusion chromatography. Mammalian cells or baculovirus-insect cells were preferentially used as expression systems, to maximize the likelihood of proper folding and glycosylation of the extracellular protein collection [10, 69]. SDS-PAGE and multiangle laser light scattering were used to analyze noncovalent aggregation and ensure high-quality protein production. Subsequently, the purified proteins were spotted on epoxysilane slides using a NanoPrint Arrayer, and protein immobilization on the microarray was determined by probing the slides with the relevant anti-tag antibodies [10]. To enhance detection of low affinity interactions, a rapid method to assemble bait proteins (whose ECD was expressed as a Fc tag-fusion protein) into multivalent complexes using fluorescently labeled protein A microbeads was developed. Proof-of-concept assays showed high sensitivity for detection of weak ePPIs characterized by micromolar *K*_*D*_, a minimal off-target binding, and more than 70% true-positive to false-positive detection ratio [10, 69]. Over the years, this extracellular protein microarray has successfully identified counterreceptors for a number of human molecules, providing relevant insights into novel pathways that coordinate a multitude of cell functions [70–72].

### 3.3. Protein Microarrays for Viral Immunomodulatory Protein Receptor Discovery

**Figure 1:**
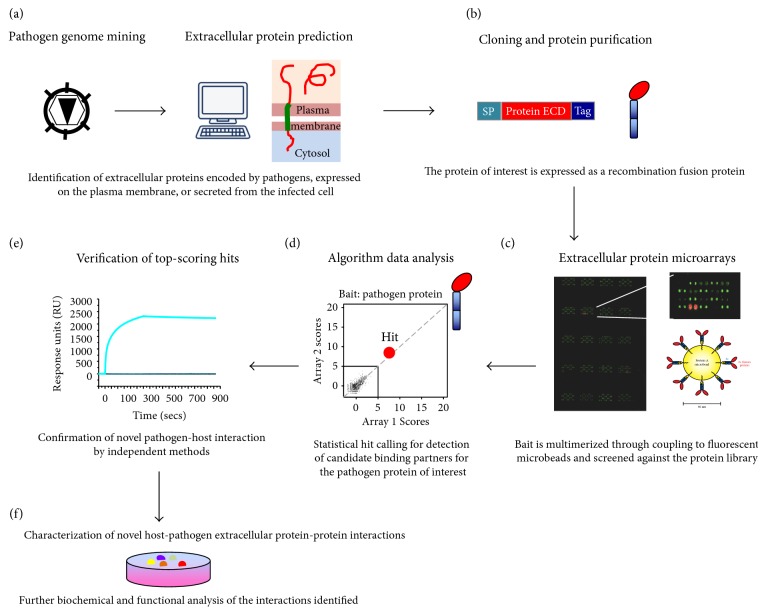
Overview of the application of the protein microarrays technology for extracellular pathogen-host protein-protein interaction discovery. (a) Identification of genes that encode for secreted factors or cell surface-expressed proteins, based on published data or bioinformatics analysis. (b) Cloning, expression, and purification of the pathogen-encoded proteins of interest. The full-length protein (secreted proteins) or the ECD (transmembrane-containing proteins) is fused to a tag for subsequent expression in the heterologous system of choice followed by affinity purification. Mammalian or baculovirus-based systems are preferred to allow for introduction of posttranslational modifications. (c) Screening of the selected pathogen-encoded proteins (baits) against extracellular human protein libraries using protein microarray technologies. Different strategies for bait multimerization have been developed to allow for detection of lower affinity interactions (see text for details). A multimerization strategy based on the coupling of Fc-tagged baits to fluorescent protein A microbeads is shown. Additional microarray-based technologies have been developed to avoid the need for extensive protein purification associated with library generation (see text for details). (d) Algorithm analysis of the protein microarray data. Frequent nonspecific binders in the human library are filtered out, and binding partners for the pathogen protein under study are depicted as high-scoring, intersecting hits. (e) Validation of the interaction between the pathogen-encoded protein of interest and the novel receptor(s) identified in the screens. Experimental validation of the protein-protein interactions may be performed using orthogonal approaches, such surface plasmon resonance, immunoprecipitation, and flow cytometry. (f) Selected binding partners may be further characterized biochemically and functionally to assess the relevance of the novel pathogen-host interactions identified. SP, signal peptide; ECD, extracellular domain.

Recently, we applied this ePPI discovery platform to the study of extracellular viral proteins (Figure 1), with a focus on human adenovirus- (HAdV-) encoded immunomodulatory proteins [28]. Despite the increasing relevance of HAdV as both pathogens and therapeutic vectors, information on the interaction of these viruses with the host immune system remains scarce [73, 74]. Interestingly, the immunomodulatory proteins encoded by these viruses, termed E3 proteins, show substantial diversity in their ECDs across and within viral species and constitute one of the most divergent regions of the HAdV genome [75, 76]. Given this striking variability, the E3 proteins have been suggested to play a role in viral tropism and pathogenesis, yet the functions of virtually all E3 proteins have remained unknown [73]. In our study, we took advantage of such unique variability to evaluate the effect of viral immunomodulatory protein diversity in extracellular host targeting. Screening of a substantial number of E3 proteins encoded by different HAdV species using the extracellular protein microarray platform allowed identification of over 50 novel virus-host interactions encompassing 5 viral species, which were fully validated by orthogonal methods [28]. These findings revealed significant diversity in extracellular host targeting and, moreover, allowed identification of semiconserved host targets, pointing towards specific human receptors that may represent previously unrecognized hubs for viral perturbation. Furthermore, most of the E3 immunomodulators were identified as multifunctional proteins, suggesting that viruses have evolved proteins capable of interfering with several cellular functions, a strategy consistent with the optimization of limited genomic resources. Such economic targeting has been often observed in intracellular targeting [15–17], but so far few examples of widespread targeting in the extracellular environment have been reported [28, 77–80], let alone a global elucidation of ePPI networks, in part due to the technical challenges associated with ePPI detection.

Remarkably, many of the HAdV E3 proteins preferentially interacted with host receptors that exert known or predicted inhibitory functions during the immune response (as defined by the presence of intracellular immunoreceptor tyrosine-based inhibitory motif, ITIM), including LILRB1 [81, 82], LAIR1 [83], and MPZL1 [84], suggesting previously unrecognized strategies of immunosuppression that may be utilized by other human pathogens [28]. Moreover, several of the receptors identified as targets for the viral proteins in this study (including the prominent cell surface molecule CD45) do not have known counterreceptors in the host, supporting the longstanding hypothesis that pathogen molecules drive the evolution of immune receptors and in many instances may represent the most relevant modulators of host receptor function [85–87]. In summary, such unbiased, microarray-based study of immunomodulatory proteins represented the first large-scale analysis of the PPI landscape of a collection of extracellular immunomodulators encoded by viruses. Future investigation of other pathogen-encoded molecules using similar extracellular protein microarrays will likely shape our understanding of the pathogen imprint in our immune system.

### 3.4. In Vitro Transcription and Translation- (IVTT-) Based Microarrays

One of the main limitations of any protein microarray platform is the lower protein coverage relative to other genome-wide methods for PPI identification, due to the costs and difficulties for generation of comprehensive protein libraries to be deposited onto the microarrays. In an attempt to address this caveat, Ramachandran and colleagues developed a method called nucleic-acid programmable protein array (NAPPA), in which DNAs are directly deposited onto the array followed by protein synthesis in situ using an in vitro transcription and translation (IVTT) system, thus avoiding the need for protein purification [88]. Although this promising approach has proven superior in generating transmembrane-containing molecules as soluble proteins, it still remains to be systematically addressed if the extracellular human proteins produced in this manner present the folding and posttranslational modifications necessary for protein functionality. Nevertheless, emerging data support the utility of NAPPA as a useful tool for the study of bacterial proteins. For example, Montor and colleagues used a bioinformatics approach to predict the* Pseudomonas aeruginosa *proteins that reside in the outer membrane of the bacteria or are secreted to the extracellular environment of the infected cell [29]. In this work, the authors utilized the NAPPA approach to screen all predicted extracellular gene products for interaction with sera from cystic fibrosis patients, where* P. aeruginosa* establish a life-threatening lung infection. From 266 bacterial proteins initially selected, 12 proteins were recognized by antibodies in the sera, indicating that these bacterial proteins represent major antigens that trigger adaptive immune responses in humans. Interestingly, robust antibody responses against three previously uncharacterized proteins were detected, suggesting this approach could help identify new extracellular proteins that exert unknown functions during the infection [29]. These results confirmed the utility of the microarrays to detect immune responses against membrane proteins encoded by pathogens, and supported the use of this methodology for diagnosis applications. In this regard, several groups have developed microarrays composed of pathogen-encoded proteins [30, 89–93]. Such pathogen protein arrays have so far being exploited mainly for diagnosis purposes, to allow screening of antibodies present in patient sera for binding to extracellular bacterial or viral antigens on the array. Nevertheless, their inherent high throughput and compatibility with multivalent bait approaches makes them a powerful tool for ePPI discovery. For example, Margarit et al. developed a* Streptococcus* microarray to find novel microbial proteins capable of binding to the human proteins fibronectin, fibrinogen, and C4BP and were able to identify a set of streptococcal proteins that interacted with these factors [31]. Nevertheless, despite such pathogen protein-based arrays offering great promise, this methodology remains to be systematically analyzed for ePPI discovery.

More recently, Yu and colleagues applied NAPPA technology in combination with the HaloTag-Halo ligand detection system to elucidate the interaction network of two effector proteins (SidM and LidA) encoded by* Legionella pneumophila*, a highly pathogenic bacteria that is the causative agent of Legionnaire's pneumonia [94]. Similarly to many pathogen proteins, these virulence factors lack significant homology to host molecules therefore complicating the assessment of their host targets and biological functions. In this work, the bacterial proteins of interest were tagged with a HaloTag, a modified haloalkane dehalogenase that covalently binds to synthetic Halo-ligands (haloalkanes) that can be fluorescently labeled, thus allowing more robust detection of bait protein binding to interactors present on the array. In this study, more than 10,000 human proteins were expressed on the NAPPA array using different IVTT techniques, leading to identification of 20 and 18 binding partners for the LidA and SidM effectors, respectively, most of them experimentally verified by pull-down [94]. Although this study focused on identification of intracellular PPI, the applicability of the NAPPA-HaloTag technology for ePPI determination should be explored in the future. Moreover, bait multimerization strategies should be implemented in order to make this approach more suitable for detection of transient PPIs.

Related to the NAPPA technology, Glick and colleagues recently built a miniaturized platform focused on human membrane proteins. By integrating the microfluidics technology, protein microarrays, and an IVTT system, this group built a new device named microfluidic-based comprehensive human membrane protein array (MPA) [32]. A notable improvement introduced by these investigators was the addition of microsomal membranes to the IVTT system to allow for improved folding and posttranslational modifications in plasma membrane proteins, both common limitations of IVTT systems. In this work, a library of 2,700 human genes encoding for membrane proteins was built and subsequently utilized to screen the large-form delta antigen (L-HDAg) encoded by the hepatitis delta virus (HDA) and whole viral particles of the simian virus 40 (SV40), a nonenveloped human pathogen. Proof-of-concept assays showed encouraging results, with over 75% true-positive rate within a small set of proteins with known interactors and, more importantly, indicated the feasibility of this approach for expression of multitransmembrane-containing proteins, a protein type that has proven challenging given their high hydrophobicity. The MPA screens identified 99 and over 150 interactions for SV40 particles and L-HDAg viral protein, respectively, and around 35 interactions were validated by coimmunoprecipitation or protein-fragment complementation assays [32]. To our knowledge, this is the first study to assess ePPIs using a comprehensive human protein library and whole viral particles (SV40) as baits, a valuable approach that may provide important insights into pathogen tropism, alongside a molecular explanation for the cell surface receptors engaged by the pathogen. Further utilization of this platform followed by a more systematic analysis of the candidate hits, including nonspecific binder determination, will be needed to assess the overall performance of the MPA technology. Regardless, this platform provides an extended version of the NAPPA approach that focuses on mammalian ePPIs and may therefore provide relevant insights into extracellular host-pathogen interactions.

The protein microarrays have represented one of the most fruitful approaches for unbiased determination of ePPIs, including host-pathogen interactions. Nevertheless, one of the main limitations of this technology is the need to generate comprehensive libraries, a process that is resource consuming and often not available to many researchers [53, 93]. Consequently, although some of the available arrays were designed to cover a significant fraction of the human proteome, any discoveries made using these platforms are limited to the proteins present in each array. The current libraries are likely to continue expanding alongside innovative approaches to facilitate sensitive detection of ePPI using protein microarray formats.

## 4. Mass Spectrometry-Based and Computational Approaches

Over the last decade, MS-based technologies have emerged as a versatile, powerful approach to decipher many aspects of the human proteome, including the characterization of protein complexes. Excellent reviews on current MS-based technologies, recent improvements, and future prospects for elucidation of PPI networks are available [95–98]. In this review, we briefly discuss the applications of some of these techniques to the study of extracellular host-pathogen interactions.

### 4.1. Mass Spectrometry-Based Characterization of Membrane Proteins and Interacting Partners

The proteins expressed on the surface of pathogens mediate functions necessary for survival, replication, immunoevasion, and transmission and therefore are logical candidates for therapeutic and vaccine design. However, the study of the surface proteome in pathogens, particularly in bacteria is constrained by the fact that commonly used prediction algorithms fail to correctly predict the location of several proteins [29, 99–101]. Despite the characterization of the extracellular proteins and their interactions still representing the Achilles heel of most proteomics methods, MS has emerged as an invaluable approach to characterize the protein composition of plasma membranes [5]. To date, several studies have exploited MS-based techniques to gain insights into the extracellular protein composition of bacterial pathogens [101–104]. For example, Palmer and colleagues studied the surface proteome of the tick-borne intracellular pathogen* Anaplasma marginale* (Rickettsiales: Anaplasmataceae) using liquid chromatography and tandem MS [105]. Interestingly, the authors found that the surface proteome of* A. marginale *isolated from tick cells, despite being less complex than that of bacteria isolated from human erythrocytes, contained a novel protein, which the authors hypothesized to play a function in human cell invasion in spite of its human counterreceptor remaining uncharacterized. This interesting observation suggests a remodeling of the bacteria surface proteome during the transition between mammalian and arthropod hosts, an aspect of the infection that could be targeted to block transmission. Similarly, several studies have pursued the identification of the proteins present in viral particles utilizing MS. Although these analyses suffer from several drawbacks associated with membrane protein characterization, particularly the poor solubility of these proteins and the low abundance of many plasma membrane proteins, these studies have revealed a complex composition for most of the viruses studied, alongside incorporation of many host proteins in the virions, in most cases with undetermined functions [106–108].

An interesting observation from some of the studies referred above is the fact that certain bacterial proteins, predicted cytoplasmic by consensus, can be found in the extracellular environment of the cell, where they may play alternative functions. In fact, the number of proteins that are secreted through noncanonical signal sequence pathways is increasingly appreciated [99, 100, 109, 110]. Little is known about these bacterial proteins originally described as cytosolic proteins but capable of exerting functions on the cell surface, which some authors have named moonlight proteins, in reference to their potential to exert multiple functions [111]. There is emerging evidence that protein moonlighting contributes to virulence of important bacterial pathogens including* Staphylococcus aureus* or* Mycobacterium tuberculosis*, sometimes in fascinating ways. For example,* M. tuberculosis* is known to encode two molecular chaperones, Cpn60.1 and Cpn60.2, which function as modulators of myeloid cells among other regulatory functions [112]. Despite these chaperones being by definition cytosolic, Cnp60.2 has been detected in significant amounts on the bacterial surface, and either recombinant Cnp60.2 or antibodies against this protein efficiently block binding of* M. tuberculosis* to macrophages [112], through a potential interaction with the receptor CD43 [113]. In addition, the protein DnaK, a Hsp70-related protein encoded by* M. tuberculosis*, can locate to the bacterial surface and functionally interact with CD40 [114] and with the HIV coreceptor CCR5 [115]. Notably, DnaK appears to block HIV binding to CCR5 in vitro, an interesting observation given the co-occurrence of* M. tuberculosis* and HIV infection [116]. Although a more detailed revision is out of the scope of this review, bacterial protein moonlighting, excellently revisited by Henderson and Martin [111], is a thought-provoking phenomenon that suggests a much more complex extracellular landscape than anticipated. Moreover, such protein moonlighting is in line with the hypothesis that pathogens have evolved multifunctional proteins as a prominent strategy for efficient use of limited genomic resources [17, 28].

Another MS-based approach that holds great promise for host-pathogen ePPI detection is the recently developed TRICEPS [33]. TRICEPS is a chemoproteomic reagent that consists of three moieties, one that binds the ligand of interest through its amino groups, a second one that binds glycosylated receptors on the cell surface, and a biotin tag for purifying the receptor peptides for subsequent identification by MS. Notably, in the initial description of the method, TRICEPS was successfully applied to the identification of receptors for extracellular ligands of diverse nature, such as secreted glycoproteins, small peptide ligands for G protein-coupled receptors, and therapeutic antibodies. Importantly, this approach has also been utilized to study cell surface molecules targeted by vaccinia virus (VACV). Interestingly, the analysis of VACV binding to HeLa cells revealed seven candidate binding partners, including the previously identified receptors AXL, chondroitin sulfate proteoglycan 4, and laminin binding protein dystroglycan 1. Further, downregulation of five out of the seven candidates using short interfering RNA reduced VACV infection by 40–60%, supporting the functionality of the interactions identified, at least in vitro [33]. Although this technology is still developing and no studies on other pathogens have been published yet, future TRICEPS-based studies promise relevant insights into pathogen interaction with distinct components of the cell surface.

### 4.2. Computational Approaches for Characterization of Pathogen-Host Interactions

As an addendum to the vast amount of knowledge acquired using MS approaches and some of the additional methodologies discussed in this review, bioinformatics offers an in silico systems biology approach that reveals a global perspective on host-pathogen interactions. Advances in computation have been fundamental to dissect the complex datasets generated in many genome-wide MS-based studies and have enabled the reconstruction of large-scale host-pathogens PPI networks, providing fundamental insights into viral disease and hence host biology [15–18, 34–36, 117]. Although the computational tools available for analysis of large datasets, in some cases developed in association with some of the high throughput screens mentioned in this review, certainly deserve a focused chapter, a couple of observations are specially notable. Commonly observed in these studies is that the intracellular viral effectors preferentially target host proteins that act as hubs (proteins with many interacting partners) or bottlenecks (proteins central to many pathways in the network) [15, 16, 36]. For example, Dyer and colleagues built a network of host-pathogen PPI by integrating published information from 190 pathogens [36]. Supporting previous findings, this analysis indicated that pathogen-encoded proteins preferentially interfere with host molecules that control critical cellular processes, such as cell death or nuclear transportation, possibly as a strategy to maximize control of the host machinery given limited genomic resources. Interestingly, this study highlighted a small set of extracellular host proteins recurrently targeted by several of the viral and bacterial pathogens analyzed, including cell surface receptors such as VEGFR2/KDR and collagen, possibly indicating previously unrecognized roles in the immune response against pathogens. Although informative, the analysis performed by Dyer and collaborators was skewed towards viruses, with a prominent enrichment in HIV strains [36]. More extensive analyses encompassing other human viruses and bacterial pathogens may reveal general strategies of immunomodulation and potential human targets suitable to therapeutic intervention. Interestingly, increasing evidence suggests that virus-host interactions are governed by principles distinct to those that dictate within-host interactions [20, 28, 85, 87, 118]. Notably, detailed analyses carried out by the Xia group highlighted significant differences between virus-host and within-host (also called endogenous) interactions, such as the tendency of viral proteins to compete with host proteins for binding to a given receptor in the absence of sequence similarity with the host counterpart or the observation that viral molecules have evolved multiple short linear motifs capable of mediating a number of diverse interactions [20, 118], features that are consistent with the multifunctional capabilities of some pathogen-encoded proteins [20, 22, 28, 77–80, 118]. Altogether, bioinformatics analysis of virus-host interactions suggest that virus-mediated targeting of host proteins is characterized by signatures of pleiotropy, economy, and convergent evolution, conclusions that are supported by emerging experimental data. Followed by thorough biological experimentation such computational-based systems biology approaches will provide a unique tool to help decipher basic global principles of pathogen-host interaction and may reveal novel ePPIs amenable to therapeutic intervention.

## 5. Genetic Screens

### 5.1. Complementary DNA Libraries and RNA-Interference-Based Approaches

Alongside protein microarrays-based technologies, MS, and computational analysis, the explosion of the functional genomics field in the last years has revolved the avenues to study pathogen interactions with their hosts, often in high throughput. In brief, genetic screens comprise gain-of-function and loss-of-function strategies, represented by complementary DNA (cDNA) libraries and RNA-interference- (RNAi-) based approaches, respectively. These methods were developed more than two decades ago and have been widely utilized by the scientific community, providing fundamental insights into the infection process. In particular, the cDNA libraries have proven extremely successful in identifying viral receptors through a gain-of-function approach, upon transduction of the cDNA library from a susceptible cell line into nonpermissive cell lines. The use of cDNA libraries is not reviewed in detail here in the interest of a more comprehensive revision of relative newer genomics-based approaches, such as the clustered regularly interspaced short palindromic repeat/CRISPR-associated protein 9 (CRISPR/Cas9) or the haploid cell screens. Nevertheless, these libraries have represented one of the most significant technologies to further our understanding of the pathogen-host interaction. For example, early studies made use of cDNA libraries to shed light on the complex mechanism exploited by hepatitis C virus for initial invasion of the cell [37–39], identified CAR as a common receptor for adenovirus 5 and coxsackievirus B [40], and were instrumental to identify SLAM1 and PVR as a receptors for measles and poliovirus, respectively [119, 120].

In turn, the RNAi technology has yielded significant insights into virus-host interactions, such as the identification of the ion transporter NRAMP as the receptor for the mosquito-borne Sindbis virus colonization of* Drosophila* cells [41]. The main power of the RNAi technology is that it allows high throughput genome-wide screens and therefore potential identification of essential factors that play roles in different aspects of the pathogen life cycle, including initial interaction with the host cell. Although RNAi screens have provided tremendous insights into host-pathogen interactions and remain widely utilized [121], inefficient gene depletion and off-target effects are important limitations of this methodology [122].

### 5.2. CRISPR/Cas9-Based Screening Technology

The increasingly popular CRISPR/Cas9 technology overcomes some of the caveats often associated with genetic manipulation and holds enormous promise for genome editing and downstream applications, including host-pathogen interaction discovery [123]. Although still early days, high throughput CRISPR/Cas9 screens for genome-wide studies have already displayed remarkable results, with high levels of genomic modification, hit confirmation, and strong phenotypic effects [124]. The development of the CRISPR/Cas9 technology has undoubtedly transformed the functional genetic analysis in mammals. Recent studies have applied the CRISPR/Cas9 technology to ablate expression of previously identified receptors for viral entry, such as the HIV coreceptors CXCR4 and CCR5, leading to resistance to infection in primary cells [125, 126]. An interesting additional application of CRISPR/Cas9 is the direct editing of viral genes important for viral fitness. This approach has recently been used to target HSV-1, CMV, and Epstein-Barr virus (EBV) essential genes, leading to a significant decrease of viral replication [127]. These studies suggest the potential use of CRISPR/Cas9 as an innovative therapeutic strategy, as aspect that will surely be further explored in the near future.

Another prominent example published recently is the identification of host factors that confer susceptibility to the evolutionary related type III secretion systems, T3SS1 and T3SS2, encoded by* Vibrio parahaemolyticus* [128]. The T3SSs are highly complex nanomachines utilized by gram-negative pathogens to inject a variable repertoire of virulence factors into the cytosol of the eukaryotic cells, enabling pathogen adhesion and internalization of modulation of host processes. Interestingly, using genome-wide CRISPR/Cas9 screens, sulfation and fucosylation of cell surface components were identified as host determinants of T3SS1- and T3SS2-mediated cytotoxicity, respectively. The authors hypothesized that interactions between sulfated cell surface molecules such as host proteoglycans and bacterial adhesins act as facilitators of T3SS1 activity, whereas fucosylated glycans on the surface may serve as receptors for T3SS2 components necessary for insertion of the complex in the host membrane [128]. The CRISPR/Cas9 approach has just started to reveal its power as a tool for unbiased identification of novel ePPIs, elegantly exemplified by the identification of CD300lf as the cell surface receptor for noroviruses, which, strikingly, was identified as the main determinant for the tropism of the murine norovirus [42]. Further optimization of this technology will unequivocally signify a tremendous advance for the discovery of extracellular host-pathogen PPIs, the processes underlying host-pathogen interactions and its possible therapeutic applications.

### 5.3. Haploid Genetic Screens

Haploid cells, in turn, allow the study of recessive phenotypes that can be masked in diploid cells, due to the difficulties of creating true genetic knockouts in mammalian cells. Despite yeast being a useful tool due to the simplicity of obtaining relevant mutants at its haploid life stage, the majority of human pathogens do not replicate in yeast therefore limiting the applicability of this approach [129]. In recent years, human haploid cells have been increasingly utilized for genome-wide loss-of-function genetic screens using insertional mutagenesis [43, 44, 47]. In initial studies, Carette and colleagues took advantage of the KBM7 cell line, a derivative of the chronic myeloid leukemia cell line (CML) with a haploid karyotype except for chromosome 8 [130]. Using gene-trap retroviruses for efficient insertional mutagenesis, the authors generated a genome-wide collection of null mutants for most nonessential genes [43]. This approach was successfully utilized to identify host factors essential for the functions of the distending toxins or CDTs, potent virulence factors secreted by a number of pathogenic bacteria. In particular, mutagenized KBM7 cells were treated with* Escherichia coli*-derived CDTs and resistant clones were isolated, leading to identification of insertions in the sphingomyelin synthase 1 and the putative G protein-coupled receptor TMEM181, suggesting that this molecule may serve as a surface receptor for the toxin [43, 44]. Similar haploid screens have identified novel receptors for a number of bacterial toxins, including the lipolysis-stimulated lipoprotein receptor for the* Clostridium difficile* transferase [45], or the low-density lipoprotein receptor-related protein 1 as a host receptor of the* Clostridium perfringens* TpeL toxin [46].

In a later study, Carette et al. generated a KBM7-derived cell line named HAP1, haploid for all chromosomes [47]. Similarly to previous studies, the authors used the retroviral gene-trap approach to mutagenize HAP1 cells followed by deep sequencing to map more than 800.000 insertions. In this study, using a replication competent vesicular stomatitis virus (VSV) carrying the Ebola virus glycoprotein, a previously unknown entry receptor for Ebola virus was identified. Notably, these haploid cell screens identified six members of the HOPS complex, proteins known to play functions in endosomal/lysosomal trafficking, as well as the Niemann-Pick C1 (NPC1) transporter as the most prominent hit of the assay. It is worth noting that NPC1 is not a surface molecule but rather an endosomal receptor. These findings led the authors to propose a novel mechanism of entry by which Ebola virus is internalized into the endocytic pathway, followed by endosome maturation and cleavage of the surface glycoprotein of the virus. Endosome fusion, mediated by the HOPS complex, would allow interaction with NCP1 containing endosomes, triggering fusion and release of the viral genome into the cytosol. Multiple cell surface receptors can lead to internalization of the Ebola virus into the endocytic pathway [131]; such redundancy in receptor usage likely explains why these receptors were not identified in the haploid cell screen [47]. Notably, independent studies have confirmed that NCP1 acts an intracellular receptor for Ebola, including a chemical screen approach, a study showing NCP1 dependence for infection of otherwise nonsusceptible cells, and more recently the elucidation of the crystal structure of this receptor bound to the Ebola virus glycoprotein [132–134].

Interestingly, after the aforementioned haploid genetic screens identified NCP1 as a noncanonical entry receptor (given its intracellular localization), other filoviruses have been shown to take advantage of this receptor [135]. The relevance of this intriguing mechanism of viral entry is further reinforced by recent work on Lassa virus, an Old World Arenavirus that, similarly to Ebola virus, causes severe to fatal hemorrhagic disease in humans [48, 136]. A genome-wide haploid screen using VSV pseudotyped with Lassa glycoprotein was performed in order to identify host factors essential for viral entry. Although *α*-dystroglycan (DAG1) was long recognized as the cell surface receptor for Lassa virus, additional factors were suspected, given the observation that certain DAG1-expressing cells are resistant to infection. The authors elegantly demonstrated that at a neutral pH, the Lassa virus glycoprotein was bound to DAG1, whereas upon exposure to lower pH (resembling the lysosome environment), a receptor switch occurred leading to strong association with the lysosomal-associated membrane protein 1 (LAMP1) [48]. Thus, similarly to Ebola virus, in the model suggested the virus would be incorporated into the endocytic pathway after interaction with its surface receptor DAG1, followed by increasingly acidic conditions that would result in interaction with LAMP1 in the lysosomal membrane, triggering membrane fusion and release of the virus in the cytosol [48].

More recently, Pillay and colleagues applied the haploid cell screening approach to the identification of host factors essential for the adeno-associated virus (AAV) serotype 2 infection, one of the leading vectors for virus-based genes therapies [49]. Notably, the most significantly enriched gene in these screens was KIAA0319L, a poorly characterized type I immunoglobulin domain-containing transmembrane protein named hereafter as the AAV receptor. Among the 46 host factors identified as hits, many were implicated in heparin sulfate proteoglycan biosynthesis as well as a number of proteins that participate in intracellular transport processes. AAV is known to attach to the cells using heparin sulfate proteoglycans and hijacks endosomal trafficking to travel to the nucleus upon invasion of the cell; thus the authors hypothesized that these additional factors may influence virus tropism [49].

Altogether, these studies elegantly demonstrate the power of genome-wide screens in human haploid cells and the power of this approach to study virus-host interactions. Future studies should further assess the applicability of this method for general detection of interactions that take place at the pathogen-plasma membrane interface. It will also be important to generate additional haploid cell lines, in order to broaden the range of pathogens and pathogen-derived molecules that can be studied using these genetic tools. In this regard, a number of haploid cell lines have been generated in mammals [137], unique tools to elucidate the basic aspects of human genetics.

### 5.4. Population Genomics for Pathogen-Host Interaction Discovery

Pathogens are among the most intriguing and prominent drivers of human evolution. Humans have adapted to the pressure imposed by microorganisms through genomic diversification, particularly through variation of genes involved in immune system function, constantly challenged by the rapidly coevolving pathogen genomes. The advent of new technologies such as next-generation sequencing and the computational tools associated have opened new avenues for the study of human genetics, making it possible to evaluate the contribution of genetic diversity to susceptibility to infection at the genomic level. The emergence of datasets of genomic variation in multiple human populations, as well as pathogen genomes, allows detection of signatures of selection, which can be exploited to identify genes with major roles in immunity (for an excellent review see [138]). Remarkably, the cell surface-expressed receptors are among the most polymorphic gene families in mammals, subjected to strong positive selection and rapid evolution, in many instances possibly driven by pathogen molecules that remain unknown [87, 139–141]. Polymorphisms in receptors and immunomodulatory genes contribute to the natural susceptibility of different individuals to infection [142–144], as illustrated by protection against HIV infection in individuals carrying homozygous polymorphisms in the viral coreceptor CCR5 [145]. The identification and further study of genes under positive selection may represent a mainstream approach to dissect novel genes involved in disease and host-pathogen interaction. A notable example is the identification of glycophorin B as the erythrocyte receptor for* P. falciparum* protein EBL-1 through examination of highly polymorphic genes in populations from malaria-endemic regions [50]. Further population genetics studies promise key insights into novel immunological mechanisms and have the potential to provide molecular details that will ultimately help design effective therapies.

### 5.5. Phagemic and Transposon Library-Based Screens

In addition to these encouraging technologies, the generation of phagemic libraries has also represented an important tool for deciphering PPIs, in this case between particular binding partners and the whole genome of specific pathogens [146–148]. Typically, pathogen-encoded molecules are expressed as fusions with phage envelope proteins, a method known as phage display that has been widely exploited to identify peptides with specific binding properties. For example, Beckmann and colleagues built a phage display library to identify novel group B streptococci proteins capable of mediating adherence to fibronectin, a major component of the extracellular matrix often exploited for colonization of the host [51]. From this analysis, the authors identified 19 genes with homology to known bacterial adhesin proteins, genes involved in virulence, transport, or metabolic processes, along with genes with uncharacterized functions. Interestingly, one of these genes showed significant homology with the ScpB protein, a peptidase found in other streptococci that inactivates the member of the human complement system C5a, suggesting that this bacterial molecule acts as a bifunctional protein, similarly to other examples of multifunctional proteins discussed above [51].

More recently, a transposon-based insertion-inactivation mutant library was elegantly utilized to identify a bacterial protein capable of targeting the surface receptor TIGIT, an inhibitory molecule present in natural killer (NK) cells and T cells [52].* Fusobacterium nucleatum* is a common oral bacterium that has been associated with colon adenocarcinoma and rheumatoid arthritis among other malignancies. In this study, Gur and colleagues showed that different strains of* F. nucleatum* blocked NK-mediated killing of human tumors. Using a library of* F. nucleatum* mutants, the authors identified Fap2 as the bacterial protein that directly interacted with TIGIT, leading to inhibition of NK cytotoxicity and downregulation tumor-infiltrating T lymphocytes activation. Immunoevasion is a hallmark of cancer; however whether members of the microbiome found within the tumor provide cancer cells with immunoregulatory properties has remained a major matter of debate [149]. These interesting findings suggest that* F. nucleatum *present in the tumor niche may enhance tumor escape by inactivating NK-mediated killing upon interaction of the fusobacterial Fap2 with the inhibitory receptor TIGIT. Of note, transposon-based mutant libraries are readily available for other pathogenic bacteria and have been successfully applied to identification of bacterial genes implicated in bacterial physiology [150–152]. It would be of interest to employ these libraries for unbiased identification of ePPIs. Notably, as mentioned above, we found that HAdV immunomodulators preferentially target other immunoreceptors that, similarly to TIGIT, also play inhibitory functions [28], suggesting this might represent a common immunosuppressive tactic evolved by pathogens. In fact, there is emerging evidence suggesting that may be the case, as a number of extracellular proteins from unrelated human pathogens have already been shown to target diverse immune receptors with inhibitory functions [28, 52, 82, 87, 153, 154]. Further exploration of inhibitory receptor targeting by other pathogens warrants exciting biological discoveries.

## 6. Concluding Remarks

Deciphering the human genome made possible the categorization of genes that encode for the human secretome; now, the challenge of the postgenomic era is to annotate the functions of those genes and their expression patterns during health and disease. A lot has been learnt from painstaking, highly focused experiments using classical biochemistry. In recent years, the impressive technological advances in proteomics, functional genomics, and computation have revolved our understanding of cell communication and function and have collectively created a versatile platform to enable biological discoveries, from mechanistic explorations to big data and systems biology analysis. Nevertheless our understanding of the molecules and mechanisms of extracellular immunomodulation and pathogen invasion remains remarkably limited.

Extracellular PPIs between host- and pathogen-encoded molecules orchestrate an enormous diversity of cellular processes, from initial colonization of the target cell to subsequent immune responses. The elucidation of these extracellular interactomes is integral to understanding the molecular basis of infection and will guide the development of more efficient or innovative therapeutics. Improvements in proteomics and genomics approaches have exponentially increased our understanding of how pathogens, particularly viruses, modulate the intracellular environment of the cell. Concomitantly, we and several other groups have implemented technologies directed towards elucidation of extracellular interactomes [2, 9, 10, 28, 32, 155], which have begun to reveal fundamental principles of extracellular host-pathogen interactions. Notably, recent studies have revealed extensive ePPI networks in model organisms such as* Drosophila* or zebrafish [2, 9]. These undertakings predict that, similarly to intracellular PPIs, extracellular networks will be highly connected, with secreted and plasma membrane-expressed proteins having multiple binding partners. However, as discussed in this review, the identification of the host factors and in many cases the pathogen molecules that mediate ePPIs have largely defied molecular identification, in part due to the technical difficulties inherent to the study of these extracellular proteins. The elucidation of the global principles dictating extracellular pathogen-host PPIs will require a coordinated effort to bring together the areas of biology and technology. There are now considerable opportunities for integrating multiple disciplines for ePPI discovery, particularly proteomics and CRISPR/Cas9 genome-wide screens, which should be powered by commensurable advances in bioinformatics and computation for big data analysis. The integration of orthogonal datasets coming from multiple “omics” approaches will be advantageous for elucidating the intricacies of the host-pathogen extracellular interactomes and will further enhance the rational identification of novel therapeutic targets by uncovering fundamental principles of biology.

The journey from classical biochemical studies towards a systems biology approach has just begun and promises major technological breakthroughs and surprising biological findings. The development of powerful technologies for ePPI discovery has already illuminated sophisticated and sometimes unexpected molecular mechanisms by which pathogens interact with their hosts and has provided unique opportunities to increase our understanding of viral and bacterial pathogenesis. Further improvement of these technologies is warranted and will surely provide the scientific community with unprecedented insights into pathogen and host biology.

## Figures and Tables

**Table 1:** Overview of the main high throughput approaches utilized to detect ePPI between pathogens and their respective hosts and relevant examples discussed thorough the text.

Pathogen	Identification method	Main findings	References
Human cytomegalovirus (hCMV)	Biochemical and MS	PDGFR*α* identified as a high affinity cell surface receptor for the CMV gHgLgO protein complex	[21]

Herpes simplex viruses (HSVs)	Biophysical	Secreted and plasma membrane-expressed glycoprotein G targets a specific set of human chemokines with high affinity	[22]

Human immunodeficiency virus type 1 (HIV)	Monoclonal antibodies	CD4 identified as the receptor for HIV infection of T cells	[23, 24]

Rhinovirus	Monoclonal antibodies	ICAM-1 as the common entry receptor for most rhinovirus serotypes	[25, 26]

*Plasmodium falciparum*	AVEXIS	BASIGIN identified as the cell-surface receptor that mediates erythrocyte infection	[27]

Human adenoviruses	Extracellular human protein microarrays	Elucidation of the extracellular interactome of adenovirus-encoded immunomodulatory proteins	[28]

*Pseudomonas aeruginosa*	Extracellular pathogen protein microarrays (NAPPA)	Screening of patient sera against all *P. aeruginosa* extracellular proteins. 12 proteins identified as potent antigens	[29]

Varicella zoster virus (VZV)	Extracellular pathogen protein microarrays (NAPPA)	Identification of 18 extracellular viral proteins that promote humoral responses upon screening of the entire VZV proteome	[30]

*Streptococcus*	Extracellular pathogen protein microarrays	Identification of new streptococcal proteins that interact with fibronectin, fibrinogen, and C4BP factors	[31]

Hepatitis delta virus LHDAg antigen	Plasma membrane microarrays (MPA)	150 candidate interactions identified between viral antigen and plasma membrane proteins	[32]

Simian virus 40 (SV40)	Plasma membrane microarrays (MPA)	99 candidate interactions between whole particles and plasma membrane proteins identified	[32]

*Vaccinia virus *(VACV)	TRICEPS (MS)	7 candidate cell surface binding partners identified for VACV	[33]

Viral pathogens	Computational studies	Insights into global principles of virus-host PPI networks	[15–18, 34–36]

Hepatitis C virus (HCV)	cDNA libraries	CD81, Claudin-1, and Occludin as cell surface receptors and some of the players involved in HSV internalization	[37–39]

Adenovirus and coxsackievirus B	Monoclonal antibodies and cDNA libraries	CAR identified as a common entry receptor for adenovirus 2/5 and coxsackievirus B	[40]

Sindbis virus	siRNA screens	NRAMP as cell surface receptor for entry into *Drosophila* cells	[41]

Murine norovirus	CRISPR/Cas9	CD300lf identified as a cell surface receptor that determines virus tropism	[42]

Bacterial distending toxins *(E. coli)*	Haploid cell screens	Sphingomyelin synthase 1 and the putative G protein-coupled receptor TMEM181 identified as toxin receptors	[43, 44]

*Clostridium difficile *and* perfringens *toxins	Haploid cell screens	Lipolysis-stimulated lipoprotein and the low-density lipoprotein receptor-related protein 1 identified as the receptors for the bacterial toxins, respectively	[45, 46]

Ebola virus	Haploid cell screens	HOPS proteins and the Niemann-Pick C1 (NCP1) transporter identified as endosomal receptors that mediate cytosol access	[47]

Lassa virus	Haploid cell screens	LAMP1, lysosomal-associated membrane protein 1 identified as an essential host factor mediating virus release to cytosol	[48]

Adeno-associated virus (AAV) serotype 2	Haploid cell screens	46 cell host factors identified, including heparin sulfate proteoglycan biosynthesis and intracellular transport genes. The immunoglobulin domain-containing transmembrane protein KIAA0319L identified as AAV receptor	[49]

*Plasmodium falciparum*	Population genomics analysis	*P. falciparum* EBL-1 protein binds to the erythrocyte receptor glycophorin B, a highly polymorphic gene in malaria-endemic regions	[50]

*Streptococcus*	Phage display	19 bacterial proteins identified as potential fibronectin-binding proteins	[51]

*Fusobacterium nucleatum*	Transposon-based mutant libraries	The bacterial protein Fap2 binds to the receptor TIGIT and downregulates NK-mediated killing of tumor cells	[52]

AVEXIS, Avidity-based extracellular interaction screen; NAPPA, nucleic-acid programmable protein array; MPA, microfluidic-based comprehensive protein array; CRISPR, clustered regularly interspaced short palindromic repeat; MS, mass spectrometry; PPI, protein-protein interaction.

## Abbreviations

AVEXIS: Avidity-based extracellular interaction screen
AAV: Adeno-associated virus
CAR: Coxsackievirus receptor
CMV: Cytomegalovirus
CRISPR/Cas9: Clustered regularly interspaced short palindromic repeat/CRISPR-associated protein 9
EBV: Epstein-Barr virus
ECD: Extracellular domain
ePPI: Extracellular protein-protein interaction
HAdV: Human adenoviruses
HCV: Hepatitis C virus
HIV: Human immunodeficiency virus
HSV: Herpes simplex virus
ITIM: Immunoreceptor tyrosine-based inhibitory motif
IVTT: In vitro transcription/translation
LAIR1: Leukocyte-associated immunoreceptor like 1
LILRB1: Leukocyte immunoglobulin-like receptor 1
MPA: Microfluidic-based comprehensive human membrane protein array
MPZL1: Myelin protein zero-like protein 1
MS: Mass spectrometry
NAPPA: Nucleic-acid programmable protein array
NCP1: Niemann-Pick C1
PDGFRa: Platelet-derived growth factor receptor alpha
PPI: Protein-protein interaction
PVR: Poliovirus receptor
SARS-CoV: Severe acute respiratory syndrome coronavirus
SV40: Simian virus 40
SLAM: Signaling-lymphocytic activation molecule
SPR: Surface plasmon resonance
T3SS: Type III secretion system
VAVC: Vaccinia virus.
